# A138 TEMPORAL CHANGES IN SURVIVAL OF METASTATIC COLORECTAL CANCER PATIENTS: A POPULATION BASED STUDY

**DOI:** 10.1093/jcag/gwab049.137

**Published:** 2022-02-21

**Authors:** C Wang, S Ghosh, O Abdelsalam, H Karachiwala

**Affiliations:** 1 Gastroenterology, University of Alberta, Edmonton, AB, Canada; 2 Cross Cancer Institute, Edmonton, AB, Canada

## Abstract

**Background:**

Colorectal cancer (CRC) remains a significant cause of morbidity and mortality in Canada. While early screening programs and improvements in surgical and oncologic therapies have led to a steady decline in the mortality rate of CRC, survival in metastatic stage IV disease remains low. Furthermore, it is unclear which factors have the largest impact on survival leading to a wide range in quoted survival times between studies.

**Aims:**

This study examines the impact of various disease, patient, and treatment factors on the outcomes and survival of stage IV CRC patients in Alberta, Canada from 2004–2014 and compares the survival data of this population to the most current statistics from clinical trials. It is hypothesized that survival should increase over the past decade and factors such as age, presence of comorbidities, number of metastases, and chemmotherapy regimen chosen will have significant impacts on overall survival.

**Methods:**

A registry-based analysis was conducted identifying patients diagnosed with advanced colorectal cancer in Alberta. Specifically, we collected demographic information for patients in 3 time intervals: 2004–2006, 2008–2010, and 2012–2014. Data collected included year of diagnosis, age, sex, number of metastatic sites at diagnosis (single vs multiple), area of residence (rural vs urban), Charlson comorbidity index, and primary site of disease. The survival data from the three timeframes were compared.

**Results:**

A total of 1476 patients were studied across the 3 time intervals. Over the three time periods examined in this study, there was essentially no clinically significant difference in the survival curves. Median survival from 2004–2006 was 22.3 months, from 2008–2010 was 24.4 months, and from 2012–2014 was 24.0 months. While median survival did increase over the 3 time periods, the improvement was not statistically significant (p=0.196). Overall survival in this cohort was 23.9 months. Patients who were >68 years old or those who had multiple sites of metastases and multiple comorbidities had a worse overall survival (p<0.05).

**Conclusions:**

Despite a decade of advances in both the surgical and medical management of Stage IV CRC, the overall survival remains low. Furthermore, there remains a wide range in the quoted survival for studies performed with this cohort. This variation is almost certainly due to the heterogeneity of the study population in stage IV disease. Future studies should employ broad inclusion criteria in order to address these important gaps in the external validity of oncology trials.

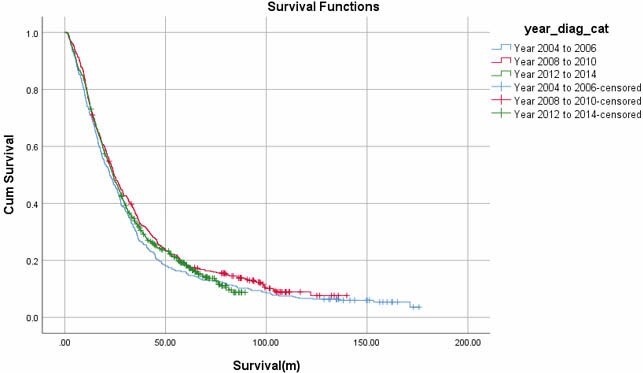

Survival of Stage IV Colorectal Cancer Over One Decade (2004–2014)

**Funding Agencies:**

None

